# Supplementary material for: Physical descriptor for the Gibbs energy of inorganic crystalline solids and temperature-dependent materials chemistry
Source: Nat Commun. 2018 Oct 9;9:4168. doi: 10.1038/s41467-018-06682-4 (PMC6177451; doi:10.1038/s41467-018-06682-4)
Supplement: Supplementary file 1 — Supplementary Information [file 41467_2018_6682_MOESM1_ESM.pdf]

Physical descriptor for the Gibbs energy of inorganic crystalline solids and temperature-dependent materials chemistry

Bartel *et al.*

Supplementary Equation 1

$$H^{\delta}(T)\left[\frac{\text{eV}}{\text{atom}}\right] = H(T) - H(298\text{ K}) \approx (2.82 * 10^{-4})T[\text{K}] - 9.13 * 10^{-2}$$

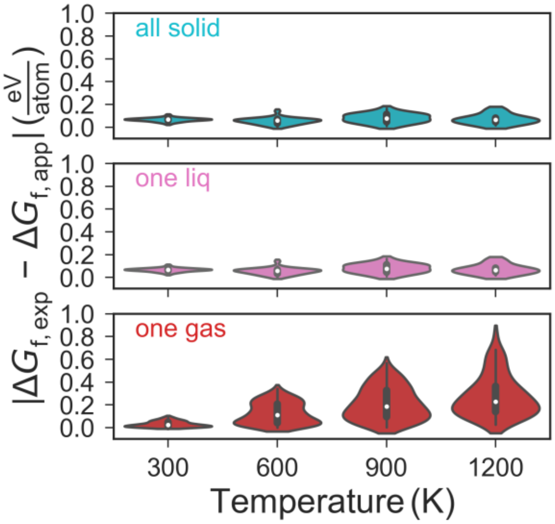

Supplementary Figure 1. Distribution of errors in assuming cancellation of solid vibrational entropy. Violin plot of absolute errors in assuming a cancellation of solid vibrational entropy between the compound and the elements comprising it. $\Delta G_f(T)$ is defined in **Equation 3**. The subscript, *app*, stands for approximation and $\Delta G_{f,app}(T)$ is defined in **Equation 4**. This figure corresponds with the bars shown in **Fig. 1c**. Each violin is a kernel density estimate of the residuals at each temperature. Within each violin is a box-and-whisker plot which provides the mean residual as a white dot, the positive and negative quartiles as the wide shaded region (box) and the minimum and maximum residual as the ends of the thin line (whisker). The violins are scaled to have constant width at each temperature.

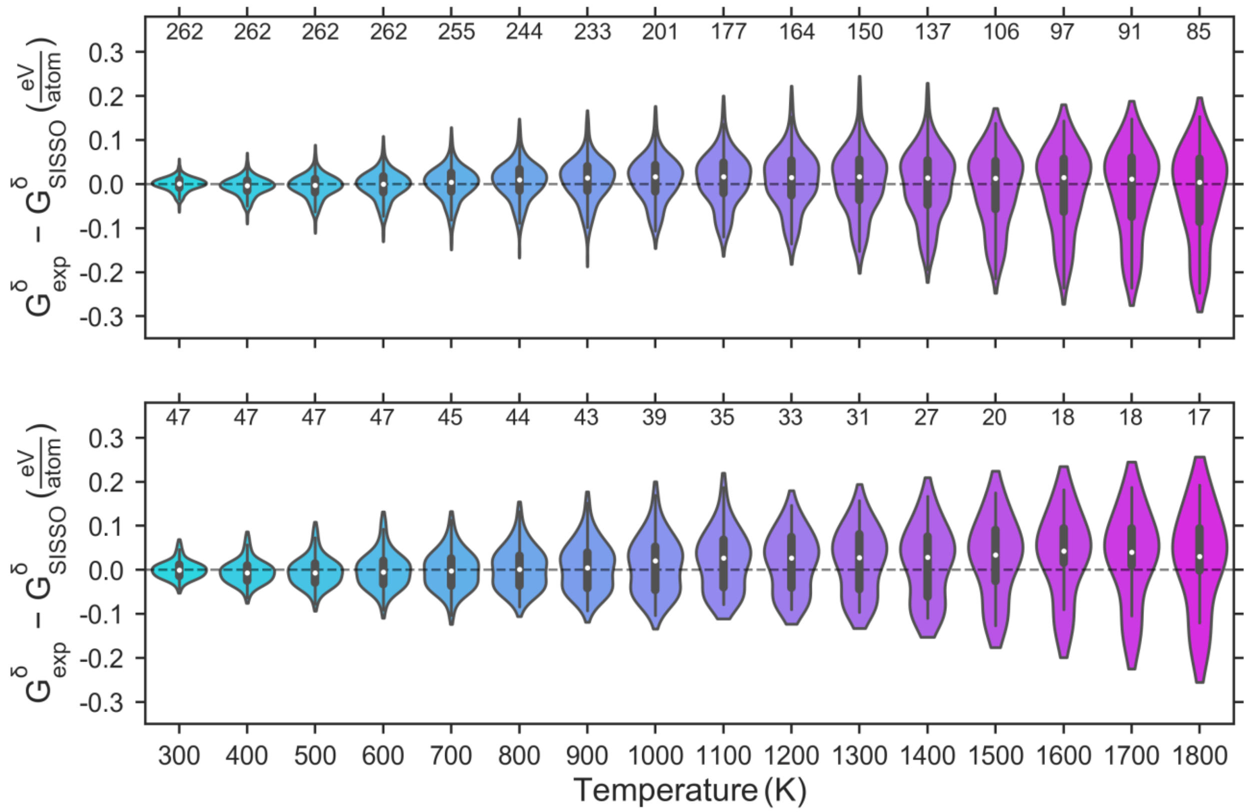

Supplementary Figure 2. Temperature-dependence of model residuals. Violin plot of residuals by temperature for the experimental dataset shown in **Fig. 2**. Above – training set; below – test set. Each violin is a kernel density estimate of the residuals at each temperature. Within each violin is a box-and-whisker plot which provides the mean residual as a white dot, the positive and negative quartiles as the wide shaded region (box) and the minimum and maximum residual as the ends of the thin line (whisker). The violins are scaled to have constant width at each temperature. The numbers at the top of each violin correspond with the number of compounds with data at each temperature. The retrieval of data at each temperature is described in "Data retrieval" in the **Methods** section.

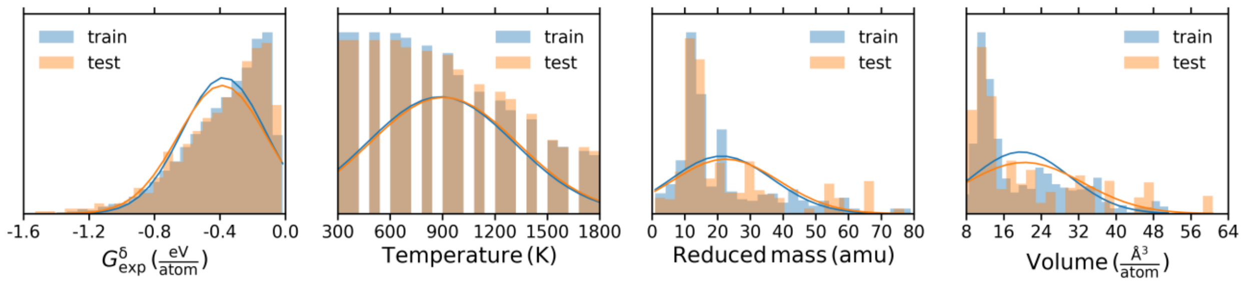

Supplementary Figure 3. Training and test set comparison. Comparing the training and test sets (**Fig. 2**) in terms of the quantities relevant to the descriptor **(Equation 3).**
