# Supplementary material for: Physical descriptor for the Gibbs energy of inorganic crystalline solids and temperature-dependent materials chemistry
Source: Nat Commun. 2018 Oct 9;9:4168. doi: 10.1038/s41467-018-06682-4 (PMC6177451; doi:10.1038/s41467-018-06682-4)
Supplement: Supplementary file 3 — Description of Additional Supplementary Files [file 41467_2018_6682_MOESM3_ESM.pdf]

Description of Additional Supplementary Files

File Name: Supplementary Data 1
Description: Compounds used for training and testing the SISSO-learned descriptor See TableS1.csv. Columns – formula = alphabetized chemical formula, source = which database contains $G\delta$, is_train = was the compound used for descriptor selection (1) or not (0), m = reduced mass (amu), V = calculated atomic volume from Materials Project (Å3 /atom), Tmax = maximum temperature with tabulated $G\delta$ (K), sg = the calculated spacegroup in Materials Project, MP_ID = the Materials Project ID for the calculation.
